# Nonhematopoietic Variants of Erythropoietin in Ischemic Stroke: Need for Step-Wise Proof-of-Concept Studies

**DOI:** 10.1100/tsw.2010.226

**Published:** 2010-11-16

**Authors:** Dirk M. Hermann

**Affiliations:** Department of Neurology, University Hospital Essen, Germany

**Keywords:** growth factor, neuroprotection, neuroplasticity, biodistribution

## Abstract

Neuroprotective, but not hematopoietic, variants of erythropoietin (EPO), such as Neuro-EPO, are promising candidates for treatment in the acute and subacute stroke phase. Characterized by its low sialic acid content and therefore exhibiting a very short plasma half-life, Neuro-EPO can probably not be administered systemically via the blood. As such, alternate routes of delivery are required. In their paper that now appears in *TheScientificWorldJOURNAL*, Rodríguez Cruz and colleagues provide evidence that Neuro-EPO promotes neurological recovery in the ischemic gerbil brain in a way that is similarly potent, if not superior, to systemically administered EPO. In view of the potential clinical use of Neuro-EPO, stringent proof-of-concept studies are urgently needed to define (1) how intranasally delivered Neuro-EPO reaches the brain, (2) which concentrations are achieved in the ischemic and nonischemic brain tissue of rodents and nonhuman primates, and (3) which are the mechanisms via which Neuro-EPO protects from injury. Only with such information should decisions be made whether intranasal Neuro-EPO may be evaluated in human patients.

Based on its potent neuroprotective[1,2] and neuronal plasticity-promoting[3] actions, the hematopoietic growth factor erythropoietin (EPO) is an attractive candidate for stroke treatment. A major advantage of this growth factor is that it is already clinically used with little side effects in patients with renal failure[4]. For this reason, experimental studies rapidly led to clinical trials in which the growth factor was administered to human patients with ischemic stroke.

Representing a large hydrophilic glycoprotein (molecular weight: 34 kDa), conventional EPO poorly crosses the blood brain barrier. The large molecular size and hydrophilicity at the moment impedes the development of a clinically applicable delivery strategy, as evidenced by the recent German multicenter EPO trial[5], which failed to detect a beneficial action despite hints of efficacy in a recent proof-of-concept trial[6].

In their paper, which appears in parallel in *TheScientificWorldJOURNAL*, Rodríguez Cruz et al.[7] have shown that Neuro-EPO, which is characterized by its low sialic acid content, exhibits more potent neuroprotective effects in a gerbil model of brain ischemia when intranasally administered than conventional EPO that was systemically applied. In behavioral tests, Neuro-EPO promoted functional neurological recovery, indicating that intranasal delivery is, if not superior, similarly potent to systemic application.

In view of its low sialic acid content, Neuro-EPO lacks hematopoietic, but still exhibits neuroprotective, activity[8,9,10]. Having a short plasma half-life of only a few minutes[11], Neuro-EPO can probably not be administered via the blood. That EPO may be applied intranasally, as shown in previous studies in rats, mice, and monkeys[12,13,14], and corroborated in the present study using a gerbil model of ischemic brain injury[7], makes Neuro-EPO attractive for preclinical use.

Important questions remain to be answered with respect to intranasal Neuro-EPO delivery. As such, the question still remains to be answered how Neuro-EPO reaches the brain tissue at all[10]. The fast appearance in the cerebrospinal fluid (CSF) of rodents and nonhuman primates[14] suggests that central nervous system passage is unrelated to axonal transport systems, as the authors correctly outline. Yet, accumulation in the CSF does not necessarily imply that the molecule also enters the brain tissue at risk. Careful biodistribution experiments will be needed to answer these questions and to determine which tissue concentrations are achieved.

Following the failure of the German multicenter EPO trial, there is a clear need for stringent proof-of-concept studies before further studies in human patients are initiated. The German multicenter EPO trial included a large percentage of thrombolyzed patients, which exhibited unexpected side effects, i.e., brain edema and hemorrhage[5], which in the meantime could be attributed to an excessive matrix metalloproteinase activation that was induced by the combined delivery of tissue-plasminogen activator (t-PA) and EPO[15]. Although data had been provided showing accumulation of EPO in the patients' CSF[6], biodistribution data on EPO are so far insufficient.

The fact that EPO should not be codelivered with t-PA, which is an established treatment of acute ischemic stroke, does not preclude the use of EPO in human patients, since the growth factor has potent plasticity-promoting actions that persist far beyond the acute stroke phase[3]. As such, the demonstration of a recovery-promoting effect of Neuro-EPO[7] is an important observation, which might suggest that neurological recovery was at least partly related to the promotion of neuronal plasticity. Future studies will have to analyze the therapeutic potential of Neuro-EPO in the postacute stroke phase and to evaluate the time window of Neuro-EPO's actions.

How should future research in this field be instructed to complement existing knowledge about the effects of nasal Neuro-EPO on structural and functional brain recovery? In view of the failure of previous neuroprotection trials, there is a clear need for biodistribution studies examining how candidate molecules accumulate in brain tissue at risk[16]. For this purpose, proof-of-concept experiments should be performed in rodents and nonhuman primates in which Neuro-EPO concentrations are quantitatively analyzed in the CSF and ischemic and nonischemic brain tissue[16]. Comparative analysis of tissue concentrations in rodents and primates should allow us to estimate the therapeutic potential of Neuro-EPO in human stroke patients and to enable dose selections for future clinical trials.

In addition, the mechanisms of action should be studied more thoroughly. We are still not aware via which receptor the neuroprotective, but not hematopoietic, variants of EPO exert their action. It has been proposed that EPO's variants induce their action via a heterodimer of the EPO receptor with a common β-receptor[17]. Final proof of evidence for this hypothesis is lacking. Furthermore, we do not know whether the mechanisms of actions of Neuro-EPO are identical to those of conventional EPO. Molecular actions of Neuro-EPO, including its effects on neuronal plasticity, should be studied more carefully in the near future, and dose-response and time-window data be provided, before clinical studies are faced. With such information, future perspectives of Neuro-EPO in the treatment of ischemic stroke are promising.
